# Clinical Features of Four West Nile Virus Cases and Its Molecular Characterization from a South Indian Tertiary Care Hospital

**DOI:** 10.1155/2020/1315041

**Published:** 2020-07-16

**Authors:** Shoba Mammen, Aiswarya Nair, Santhosh Kumar, Zayina Zonderveni, A. T. Prabhakar, Turaka Vijay Prakash, Sanjith Aaron, Mathew Alexander, Anand Zachariah, Asha Mary Abraham

**Affiliations:** ^1^Department of Clinical Virology, Christian Medical College, Vellore, Tamil Nadu, India; ^2^Department of Medicine, Christian Medical College, Vellore, Tamil Nadu, India; ^3^Department of Neurology, Christian Medical College, Vellore, Tamil Nadu, India

## Abstract

West Nile virus (WNV) is currently a significant reemerging virus of the 21st century. It belongs to the family Flaviviridae and genus *Flavivirus*. Although it is primarily transmitted by the *Culex* spp of mosquitoes, other routes of transmission are also well defined. Of eight lineages described, Lineage 1a has been reported from many parts of South India and is known to cause neuroinvasive illness. Many tests and serological techniques have been described to diagnose WNV infection such as complement fixation, neutralization, heamagglutination inhibition, ELISA, and PCR for molecular confirmation. The latter far outweighs the limitations inherent in the other tests. WNV infection is being reported from Vellore for the first time after 1968. This paper aims to describe four cases of WNV infection causing central nervous system manifestations with its molecular characterization. West Nile virus infection was diagnosed with the available molecular techniques such as PCR and sequencing, which emphasizes the need for considering West Nile virus in the differential diagnosis of acute meningoencephalitis and the wider availability of molecular diagnostic tests.

## 1. Introduction

WNV, an emerging flavivirus, discovered 8 decades ago, is being increasingly reported from all over the world. Historically, West Nile virus was first described from the West Nile province of Uganda in 1937 [1]. Furthermore, in 1957, the first neuroinvasive disease due to WNV was reported in Israel [2]. Through the course of the years 1950s through 1980s, West Nile outbreaks associated with mild febrile illness were reported from Israel, Egypt, India, France, and South Africa [2–4]. West Nile outbreaks were reported from all the continents globally. It came to the limelight after an outbreak in New York State, USA, in 1999 caused by the NY99 strain [5]. In India, seropositivity to West Nile virus in human beings was mentioned since 1952 in the western states of India. Smithburn et al. in 1954 described how sera collected from 38 localities in India and tested against 15 arthropod-borne viruses showed West Nile neutralization in 35% of sera [6]. Subsequently, cases due to WNV infection were reported from all parts of India from 1968 [7] to 2012 [8].

WNV is transmitted by the bite of *Culex* mosquitoes. Passerine birds act as reservoir hosts, and horses and men are dead-end hosts developing low levels of viremia [9].Other mosquito spp such as *Aedes anopheles* [10] and ticks [11] were also found to harbor WNV. WNV was also identified in other animal species such as frugivorous bats [12], wild birds [13], and pigs [14].

The other important routes of transmission of WNV are blood transfusion, organ transplantation, mother-to-child transplacental transmission, and breast milk [15] and as an occupational hazard in lab workers through percutaneous inoculation [16] and airborne route [17]. However, human-to-human or nonhuman vertebrate-to-human transmission has not been recorded till date [18].

Most WNV infections are subclinical. The spectrum of West Nile illness ranges from asymptomatic to fatal neurological illness, with <1% resulting in fatal encephalitis, meningitis, and acute poliomyelitis-like illness [1].The incidence of neuroinvasive illness increases with age, diabetes, and immunosuppression. This may be attributed to the disruption of the cerebral endothelium in the case of hypertension and cerebrovascular disease. An increase in viral load and duration of viremia can occur in immune senescence and immunosuppression [18].

Phylogenetically, WNV has been classified into 8 major lineages of which lineage 1a and lineage 2a are the most significant human pathogens [19]. All continents have reported cases of West Nile infection by lineage 1. Until the early 2000s, West Nile virus lineage 2 was restricted to sub-Saharan Africa. Later, it spread to parts of central and Eastern Europe such as Hungary and southern Russia as human and animal outbreaks [20].

In this report, we describe the first four lab-confirmed cases of WNV in Vellore district, Tamil Nadu, South India, since 1968.

Four patients in the study had West Nile Virus positivity in CSF PCR testing. The case descriptions are as follows:

All three cases were from Vellore district. The first case presented to us in early August 2015 and both the other cases in September 2015, while the last case presented in April 2017.

## 2. Case Presentation

### 2.1. Case 1

A 35-year-old woman from north Tamil Nadu presented to medical outpatient department in August 2015 with fever of one-day duration and 3 episodes of generalized tonic-clonic seizures. She was fully conscious at the time of presentation. Physical examination did not reveal any neck stiffness or focal deficits. CSF examination revealed 7 lymphocytes and normal protein and glucose. She had mild elevation of transaminases, SGOT of 136 mg/dl, and SGPT of 65 mg/dl. CT brain showed a normal pattern. Magnetic resonance imaging was not performed. The patient was discharged from hospital after two days, with no residual sequelae, and remained asymptomatic at 1-month follow-up. Clinical diagnosis was acute meningoencephalitis.

### 2.2. Case 2

A 28-year-old man from north Tamil Nadu presented in September 2015 with a history of fever, headache, and altered sensorium for one day. There was no history of seizures. Physical examination did not reveal any neck stiffness or focal deficits. His GCS was 14/15. CSF examination revealed 70 lymphocytes, normal CSF glucose, and a CSF protein elevation of 58 mg/dl. CT brain and MRI were normal. The patient was discharged from hospital after four days, with no residual sequelae. He was asymptomatic at 1-month follow up. Clinical diagnosis was acute meningoencephalitis.

### 2.3. Case 3

A 24-year-old woman from north Tamil Nadu presented in September 2015 with fever, headache of 3-day duration, and an episode of generalized tonic-clonic seizure. There was no history of altered sensorium. Physical examination showed focal onset seizure in the left upper limb with secondary generalization. She was conscious and oriented. There was no neck stiffness or focal deficits. CSF examination showed 20 WBCs with 92% neutrophils, normal glucose, and an elevated CSF protein (216 mg/dl). CT brain showed raised intracranial tension. MRI showed gyral edema in the left parietal region and minimal meningeal enhancement. Her clinical condition improved; however, she developed new onset abnormal movements involving her trunk and limbs after 7 days which were different from the seizures at hospital admission. After detailed history and evaluation, the same were diagnosed to be pseudoseizures. She had a significant past history of suicidal attempts and psychiatric behaviour. The patient was discharged from hospital after 16 days with no residual sequelae. She was followed up after 1 month at which time she was asymptomatic. Clinical diagnosis was acute meningoencephalitis.

### 2.4. Case 4

A 16-year-old high school student from north Tamil Nadu presented to the neurology outpatient department in April 2017 with a history of high-grade intermittent fever and holocranial headache, which was followed a day later by asymmetrical weakness in all four limbs including the bulbar muscles. Clinical examination showed exaggerated deep tendon reflexes including the jaw jerk with bilateral extensor plantar reflexes. He had an upper motor neuron type of facial involvement on his right side and expressive aphasia. His symptoms progressed and in another 3 days, he became bed bound, unable to swallow or verbalize, but was able to communicate with his parents only through eye movements and sounds. Subsequently, his sensorium worsened and he had to be intubated and mechanically ventilated. CSF showed normal counts and sugars with elevated protein. MRI showed symmetric swelling of the thalamus, hypothalamus, brainstem, and cerebellar hemispheres with areas of patchy central diffusion restriction. Also, there was diffuse spinal cord involvement showing long segment cord swelling in the cervical cord as well as in the lower thoracic cord along with conus. The electromyography showed features of motor axonopathy involving all limbs and paraspinal muscles, suggesting significant lower motor neuron involvement.

#### 2.4.1. Clinical Diagnosis

Clinical diagnosis was acute necrotising encephalomyelitis with peripheral nervous system involvement. The patient was started on IV acyclovir, which was later changed to oseltamivir when he was found to be influenza H1N1-positive. He was also administered a course of IVIg (IV immunoglobulin). The patient gradually improved and was weaned off the ventilator. He underwent a rehabilitation programme and was discharged after 40 days. At the time of discharge, he was walking with one-person support and was only minimally dependent for his activities of daily living. He was followed up in our outpatient clinic. He continued to improve and had achieved a modified Rankin Score of 1 after 3 months of discharge.

**Figure 1:**
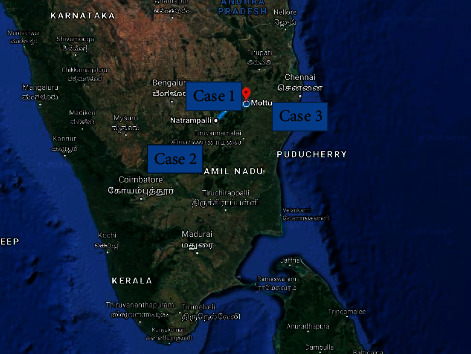
Geographical location of West Nile encephalitis cases (Case 1–Case 3).

The first three cases were from Vellore district. They presented on 11 September 2015 (Case 1), 8 August 2015 (Case 2), and 22 September 2015 (Case 3) as shown in Figure 1 for geographical location of cases.

## 3. Laboratory Diagnosis

In all the four cases, CSF was collected by lumbar puncture under sterile aseptic conditions and was transported at 2–8°C to the Department of Clinical Virology. CSF was tested for the presence of West Nile RNA by two conventional PCRs and one real-time PCR. RNA was extracted from the CSF samples according to the manufacturer's instructions using the QIAmp viral RNA mini kit (Qiagen, Germany).

**Figure 2:**
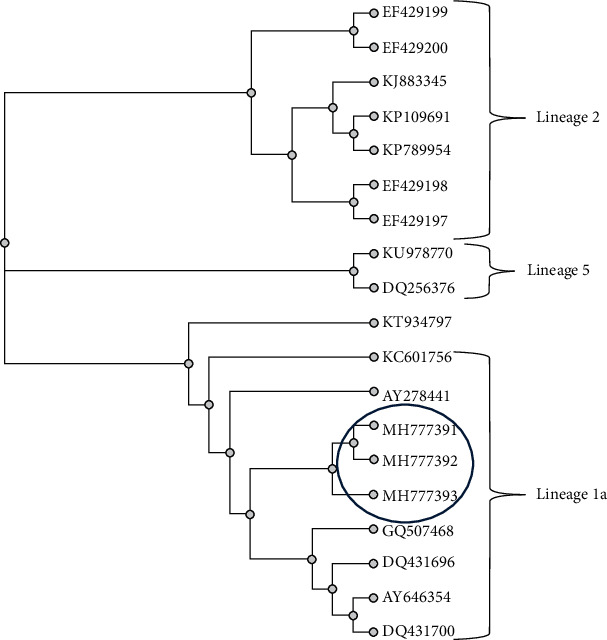
The evolutionary history was inferred using the neighbor-joining method. The optimal tree with the sum of branch length = 0.37. The number of replicate trees in which the associated taxa clustered together in the bootstrap test was 1000. The tree is drawn to scale, with branch lengths in the same units as those of the evolutionary distances used to infer the phylogenetic tree. The evolutionary distances were computed using the maximum composite likelihood method and are in the units of the number of base substitutions per site. All positions containing gaps and missing data were eliminated. There were a total of 201 positions in the final dataset. Evolutionary analyses were conducted in MEGA7.

After the conversion of RNA to cDNA, a conventional in-house PCR targeting the E gene was performed. Positive specimens showed a PCR band at the 70 bp region along with amplification of the in-house control NY99 strain at the 70 bp region. Cloning of the isolate was performed using the TOPO TA cloning kit (Invitrogen, Carlsbad, California, 92008) followed by presequencing, sequencing, and postsequencing clean-up steps. The sequence was subjected to Blast (NCBI) and confirmed as WNV. The conventional in-house PCR as described earlier (targeting the *env* gene) gave a PCR band at the 201 bp region [8]. This was subjected to sequencing and phylogenetic analysis, which indicated that these strains were of lineage 1a. A real-time PCR as previously described targeting the E gene amplified three isolates, while the in-house control NY99 strain was amplified [21]. All these sequences were aligned and submitted to GenBank. The GenBank accession numbers are shown circled in Figure 2. Of the four isolates, one isolate was not sequenced due to lack of availability of the sample for sequencing.

## 4. Discussion

West Nile virus is being increasingly reported in the present decade from India, especially the southern states. In 2011, an acute encephalitic syndrome involving 208 cases was reported from Kerala, South India. Of these, three cases causing acute flaccid paralysis were confirmed as West Nile virus infection by microneutralization assay. A seropositivity of 76.19% due to West Nile was demonstrated by serum neutralization assay [22]. In 2012, it was additionally reported from south Tamil Nadu, as a viral fever outbreak with multifocal retinitis involving 27 cases confirmed by molecular techniques [20]. There were 3 cases of acute flaccid paralysis due to West Nile encephalitis reported from Kerala in 2013 presenting as locked-in state and flaccid paralysis (1 case) and asymmetric paraparesis (2 cases) [23]. The recent report of death of a child in Mallapuram in Kerala due to West Nile virus encephalitis on March 18, 2019 indicates that there is ongoing West Nile transmission.

We also report four molecularly confirmed cases of West Nile virus causing acute neurological illness.

Of the 8 lineages of WNV described in the literature, only lineages 1 and 2 comprise the most important human pathogens. The most important neuropathogen is lineage 1a, being widespread globally and described in most continents [24]. Lanciotti described partial genome analysis of the Indian isolates of WNV on the basis of partial E or NS5 sequences as belonging to genetic lineage 1 (clade1c). Bondre et al. have reclassified the previously described lineage 1c as lineage 5 [4, 25, 26]. Outbreaks of West Nile virus due to lineage 1a were described in 2011 [22], 2012 [20], and 2013 [27] in South India. Similarly, our isolates are also phylogenetically classified as lineage 1a.

Molecular testing combined with serology is the mainstay of laboratory diagnosis of WNV infection. Serological cross reactivity with dengue virus, chikungunya virus, and Japanese encephalitis virus is the main limitation; hence, diagnosis with a specific IgM capture ELISA kit is problematic. This is especially true in the southern states of India where dengue, chikungunya, and JE are endemic and have seasonal outbreaks after monsoon. The confirmatory diagnosis would be detection of specific neutralizing antibodies using the plaque reduction neutralization test. This is a very cumbersome, laborious, and time-consuming test carried out in reference laboratories by trained personnel [22, 28]. Serum IgM may not indicate a current illness due to West Nile virus since it is known to persist for as long as 1 year [29].

The viremic period of WNV averages 6.9 days. Hence, the nucleic acid amplification from CSF and serum is demonstrated for only a maximum of 7 days [28]. Three of the 4 CSF samples were confirmed WNV PCR positive by two conventional PCRs and one real-time PCR; the fourth (from patient 3) did not amplify in the real-time PCR (this may be attributed to multiple freeze-thaws of the sample). Although in the literature, molecular identification using PCR is described as having limited utility due to the short viremic period and the low sensitivity of TaqMan PCR [17], all four of our cases were molecularly confirmed within four days of onset of symptoms.

Of the four confirmed cases, three cases had a history of fever and headache as prodromal symptoms, which is quite common as described in the literature [17]. Although seizures are described as uncommon in West Nile virus infection [30, 31], Case 1 and Case 3 of our series had generalized tonic-clonic seizures. This is similar to a study described in 2015 from Sri Lanka [32]. None of the cases had history of diarrhoea, rash, or vomiting as quoted in the literature. Another finding was that of facial nerve palsy, occurred in Case 4 only, which has been previously described in the literature as occurring in 13% of cases [33]. Flaccid paralysis was seen in Case 4 only, as quoted in the literature as developing an acute onset or rapidly progressive asymmetric flaccid weakness [34]. The third case had CSF neutrophilia, while the second case had lymphocytic predominance as described in the literature with normal sugars and elevated protein in majority of cases [35]. All the four reported cases, especially the first three cases, completely recovered without any residual sequelae akin to the study described from Sri Lanka in 2015 [32]. In another two studies conducted in 2000 and 2001 in the United States, 37% of the patients recovered fully [36, 37]. Significant lower motor neuron involvement in Case 4 suggests encephalomyelitis due to West Nile infection. In the epidemic of West Nile infection in New York City in 1999 in which there were 59 cases and 7 deaths, a community study demonstrated that there were probably 8200 (range 3500–13000) cases of West Nile virus infections [38]. They estimated that about 2.6% of the population was infected and for every one patient with meningoencephalitis, 30 people were infected. In the literature, there have been 12 outbreaks of West Nile encephalitis [39].

The present study showed that there was a cluster of 3 cases from Vellore district in the months of August and September of 2015. This suggests that the three cases of meningoencephalitis represented the tip of the iceberg of an epidemic of West Wile infection. The first three cases presented with an acute meningoencephalitis picture and in none of them was the diagnosis of West Nile encephalitis considered in the differential diagnosis. The infection was diagnosed because of the availability of molecular testing. This emphasizes the importance of considering West Nile virus in the differential diagnosis of acute meningoencephalitis and the wider availability of molecular diagnostic tests.

In Carey et al.'s study of 1968, acute-phase serum specimens were collected daily from a total of 396 episodes of fever cases from patients with respiratory infections, diarrhoea, and even central nervous system disease. Isolates were identified by means of complement fixation (CF), hemagglutination inhibition (HI), and/or neutralization. One case of West Nile virus infection was identified by both HI and CF results. This was a case of a seven-year-old girl from rural North Arcot district, who presented with four days of fever and convulsions proceeding to coma. The child regained consciousness but developed a left facial palsy [7]. Since 1968, when the West Nile virus was reported using older techniques such as complement fixation and hemagglutination, this is the first time in Vellore district, South India, that West Nile virus has been confirmed by molecular techniques. The study suggests that there could be a reservoir of West Nile infections which remain in the bird species of Vellore district which is being transmitted to human population during epidemic outbreaks. It is not clear if these cases represent a resurgence of West Nile infection in Tamil Nadu or whether West Nile infection has been present but is now being diagnosed due to the availability of molecular techniques.

West Nile virus is a reemerging virus, increasingly reported from India. It has been reported from various parts of South India causing acute flaccid paralysis in 2011 and ocular retinitis in 2012. The report shows the detection of four cases of West Nile meningoencephalitis in Vellore after 47 years. These cases suggest that there may be a local reservoir and more widespread transmission in the community. Physicians need to consider it as a differential diagnosis of acute meningoencephalitis. In-house PCRs developed in secondary care centers will be useful in confirming the diagnosis of West Nile virus infection and documenting its epidemiology. These cases suggest the need for suitable local strategies for vector control and prevention of transmission.

## References

[1] Smithburn K. C., Hughes T. P., Burke A. W., Paul J. H. (1940). A neurotropic virus isolated from the blood of a native of Uganda 1. *The American Journal of Tropical Medicine and Hygiene*.

[2] Spigland I., Jasinska-Klingberg W., Hofshi E., Goldblum N. (1958). Clinical and laboratory observations in an outbreak of west nile fever in Israel in 1957. *Harefuah*.

[3] Balanca G., Gaidet N., Savini G. (200). Low west nile virus circulation in wild birds in an area of recurring outbreaks in Southern France. *Vector-Borne and Zoonotic Diseases*.

[4] Bondre V. P., Jadi R. S., Mishra A. C., Yergolkar P. N., Arankalle V. A. (2007). West nile virus isolates from India: evidence for a distinct genetic lineage. *Journal of General Virology*.

[5] Asnis D. S., Conetta R., Teixeira A. A., Waldman G., Sampson B. A. (2000). The west nile virus outbreak of 1999 in New York: the flushing hospital experience. *Clinical Infectious Diseases*.

[6] Smithburn K. C., Kerr J. A., Gatne P. B. (1954). Neutralizing antibodies against certain viruses in the sera of residents of India. *Journal of Immunology*.

[7] Carey D. E., Rodrigues F. M., Myers R. M., Webb J. K. (1968). Arthropod-borneviral infections in children in Vellore, South India, with particular reference to dengue and west nile viruses. *Indian Pediatrics*.

[8] Shukla J., Saxena D., Rathinam S. (2012). Molecular detection and characterization of west nile virus associated with multifocal retinitis in patients from Southern india. *International Journal of Infectious Diseases*.

[9] Petersen L. R., Brault A. C., Nasci R. S. (2013). West nile virus: review of the literature. *Journal of the American Medical Association*.

[10] Gubler D. J. (2007). The continuing spread of west nile virus in the western hemisphere. *Clinical Infectious Diseases*.

[11] Lawrie C. H., Uzcátegui N. Y., Gould E. A., Nuttall P. A. (2004). Ixodid and argasid tick species and west nile virus. *Emerging Infectious Diseases*.

[12] Paul S. D., Rajagopalan P. K., Sreenivasan M. A. (1970). Isolation of the west nile virus from the frugivorous bat, Rousettusles chenaulti. *Indian Journal of Medical Research*.

[13] Mishra N., Kalaiyarasu S., Nagarajan S. (2012). Serological evidence of west nile virus infection in wild migratory and resident waterbirds in eastern and northern India. *Comparative Immunology, Microbiology and Infectious Diseases*.

[14] Ratho R. K., Sethi S., Prasad S. R. (1999). Prevalence of Japanese encephalitisand west nile viral infections in pig population in and around Chandigarh. *Journal of Communicable Diseases*.

[15] Sampatkumar P. (2003). West nile virus: epidemiology, clinical presentation, diagnosis, and prevention. *Mayo Clinic Proceedings*.

[16] Centers for Disease Control Prevention (2002). Laboratory-acquired west nile infections- United States, 2002. *MMWR Morbidity and Mortality Weekly Report*.

[17] Nir Y. D. (1959). Airborne west nile virus infection. *The American Journal of Tropical Medicine and Hygiene*.

[18] Campbell G. L., Marfin A. A., Lanciotti R. S., Gubler D. J. (2002). West nile virus. *The Lancet Infectious Diseases*.

[19] Bakonyi T., Ivanics É., Erdélyi K. (2006). Lineage 1 and 2 strains of encephalitic west nile virus, central Europe. *Emerging Infectious Diseases*.

[20] Hubalek Z., Halouzka J. (1999). West nile fever-a reemerging mosquito-borne viral disease in Europe. *Emerging Infectious Diseases*.

[21] Lanciotti R. S., Kerst A. J., Nasci R. S. (2000). Rapid detection of west nile virus from human clinical specimens, field-collected mosquitoes, and avian samples by a taqman reverse transcriptase-PCR assay. *Journal of Clinical Microbiology*.

[22] Anukumar B., Sapkal G. N., Tandale B. V., Balasubramanian R., Gangale D. (2014). West nile encephalitis outbreak in Kerala, India, 2011. *Journal of Clinical Virology*.

[23] Maramattom B., Philips G., Sudheesh N., Arunkumar G. (2014). Acute flaccid paralysis due to west nile virus infection in adults: a paradigm shift entity. *Annals of Indian Academy of Neurology*.

[24] Rizzoli A., Jimenez-Clavero M. A., Barzon L. (2015). The challenge of west nile virus in Europe: knowledge gaps and research priorities. *Eurosurveillance*.

[25] Lanciotti R. S. (1999). Origin of the west nile virus responsible for anoutbreak of encephalitis in the Northern US. *Science*.

[26] Lanciotti R. S., Ebel G. D., Deubel V. (2002). Complete genome sequences and phylogenetic analysis of west nile virus strains isolated from the United States, Europe, and the Middle East. *Virology*.

[27] Balakrishnan A., Butte D. K., Jadhav S. M. (2013). Complete genome sequence of west nile virus isolated from Alappuzha district, Kerala, India. *Genome Announcements*.

[28] Busch M. P., Kleinman S. H., Tobler L. H. (2008). Virus and antibody dynamics in acute west nile virus infection. *The Journal of Infectious Diseases*.

[29] Roehrig J. T., Nash D., Maldin B. (2003). Persistence of virus-reactive serum immunoglobulin M antibody in confirmed west nile virus encephalitis cases. *Emerging Infectious Diseases*.

[30] Chowers M. Y., Lang R., Nassar F. (2001). Clinical characteristics of the west nile fever outbreak, Israel, 2000. *Emerging Infectious Diseases*.

[31] Ceausu E., Erscoiu S., Calistru P. (1997). Clinical manifestations in the west nile virus outbreak. *Romaninan Journal of Virology*.

[32] Lohitharajah J., Malavige G. N., Chua A. J., Ng M. L., Arambepola C., Chang T. (2015). Emergence of human west nile virus infection in Sri Lanka. *BMC Infectious Diseases*.

[33] Tyler K. L. (2004). West nile virus infection in the United States. *Archives of Neurology*.

[34] Li J., Loeb J. A., Shy M. E. (2003). Asymmetric flaccid paralysis: a neuromuscular presentation of west nile virus infection. *Annals of Neurology*.

[35] Tyler K. L., Pape J., Goody R. J., Corkill M., Kleinschmidt-DeMasters B. K. (2006). CSF findings in 250 patients with serologically confirmed west nile virus meningitis and encephalitis. *Neurology*.

[36] Nash D., Mostashari F., Fine A. (2001). The outbreak of west nile virus infection in the New York city area in 1999. *New England Journal of Medicine*.

[37] Weiss D., Carr D., Kellachan J. (2001). Clinical findings of west nile virus infection in hospitalized patients, New York and New Jersey, 2000. *Emerging Infectious Diseases*.

[38] Mostashari F., Bunning M. L., Kitsutani P. T. (2001). Epidemic west nile encephalitis, New York, 1999: results of a household-based seroepidemiological survey. *The Lancet*.

[39] Solomon T., Ooi M. H., Beasley D. W., Mallewa M. (2003). West nile encephalitis. *BMJ*.

